# Concordance between stabilizing sexual selection, intraspecific variation, and interspecific divergence in *Phymata*

**DOI:** 10.1002/ece3.2537

**Published:** 2016-10-12

**Authors:** David Punzalan, Locke Rowe

**Affiliations:** ^1^ Department of Natural HistoryRoyal Ontario MuseumTorontoONCanada; ^2^ Department of Ecology and Evolutionary BiologyUniversity of TorontoTorontoONCanada

**Keywords:** adaptive landscape, constraint, covariance matrix, D‐matrix, stasis

## Abstract

Empirical studies show that lineages typically exhibit long periods of evolutionary stasis and that relative levels of within‐species trait covariance often correlate with the extent of between‐species trait divergence. These observations have been interpreted by some as evidence of genetic constraints persisting for long periods of time. However, an alternative explanation is that both intra‐ and interspecific variation are shaped by the features of the adaptive landscape (e.g., stabilizing selection). Employing a genus of insects that are diverse with respect to a suite of secondary sex traits, we related data describing nonlinear phenotypic (sexual) selection to intraspecific trait covariances and macroevolutionary divergence. We found support for two key predictions (1) that intraspecific trait covariation would be aligned with stabilizing selection and (2) that there would be restricted macroevolutionary divergence in the direction of stabilizing selection. The observed alignment of all three matrices offers a point of caution in interpreting standing variability as metrics of evolutionary constraint. Our results also illustrate the power of sexual selection for determining variation observed at both short and long timescales and account for the apparently slow evolution of some secondary sex characters in this lineage.

## 1 Introduction

Evolution of complex phenotypes is often modeled in a quantitative genetic framework, whereby intergenerational change in trait distributions depends on the joint influence of phenotypic selection and genetic covariance. The essential components of this framework are readily parameterized, and these have contributed substantially to our understanding of the strength and form of contemporary selection (Endler, 1986; Kingsolver et al., 2001), patterns of standing genetic variance (Houle, 1992; Mousseau & Roff, 1987), as well as rates of short‐term evolution (Hendry & Kinnison, 1999). Yet, the utility of these parameters to predict (or explain) evolution over the long‐term is contentious and the prospect of connecting micro‐ to macroevolution remains a central challenge (Arnold, Bürger, Hohenlohe, Ajie, & Jones, 2008; Arnold, Pfrender, & Jones, 2001; Bégin & Roff, 2004; Charlesworth, Lande, & Slatkin, 1982). This aspect of predictability is the cornerstone of an open empirical question in evolutionary biology: the relative importance of genetic constraints on dictating the direction and extent of evolutionary change (Agrawal & Stinchcombe, 2009; Blows & Hoffman, 2005; Conner, 2012; Hansen & Houle, 2004, 2008).

Two related empirical observations at the macrolevel have frequently been cited as evidence of persistent genetic constraints. First, long periods of stasis (i.e., little or bounded evolutionary change in a given trait) appear to be the overwhelming mode of phenotypic evolution (Estes & Arnold, 2007; Gingerich, 2001; Uyeda, Hansen, Arnold, & Pienaar, 2011). Given the typically strong contemporary directional selection estimated for wild populations (Hoekstra et al., 2001; Kingsolver et al., 2001), one possible interpretation is that there is often a lack of genetic variance in the direction of selection (Blows & Hoffman, 2005; Kingsolver & Diamond, 2011). Second, it is often observed that the codistribution of trait means among closely related species (i.e., the divergence matrix, **D**) bears resemblance to standing phenotypic (**P**) and/or genetic (**G**) trait covariances observed within a species (Baker & Wilkinson, 2003; Blows & Higgie, 2003; Bolstad et al., 2014; Chenoweth, Rundle, & Blows, 2010; Hunt, 2007; Kluge & Kerfoot, 1973; Kolbe, Revell, Szekely, Brodie, & Losos, 2011; Lofsvold, 1988; Revell, Harmon, Langerhans, & Kolbe, 2007; Schluter, 1996). Proportionality between intraspecific covariation and interspecific covariation is suggestive of macroevolutionary divergence constrained to occur along “genetic lines of least resistance” and in a manner predicted under a neutral model of evolution by mutation and drift (Lande, 1979; Lynch & Hill, 1986; Schluter, 1996).

It is important to recognize, however, that concordance between intra‐ and interspecific covariance matrices is not a prediction exclusive to a “genetic constraint” hypothesis and the key to understanding the resemblance between these two matrices might actually be found in a third matrix, **γ**, summarizing the pattern of nonlinear selection on single traits or on trait combinations (Lande & Arnold, 1983; Phillips & Arnold, 1989). The evolution of genetic covariances after a single generation of selection depends largely on nonlinear selection (Lande, 1980; Phillips & McGuigan, 2006), and, ultimately, standing trait variance is predicted to align with **γ** (Cheverud, 1984; Jones, Arnold, & Bürger, 2003, 2004; Melo & Marroig, 2015; Zeng, 1988). Theoretically, the evolution of genetic covariances need not be reflected in the **P**‐matrix (Roff, Prokkola, Krams, & Rantala, 2012; Willis, Coyne, & Kirkpatrick, 1991); however, empirical treatments have often found similarities between genetic and phenotypic covariances (Cheverud, 1988, 1996; Hohenlohe & Arnold, 2008; Marroig & Cheverud, 2001; Roff, 1995; Steppan, Phillips, & Houle, 2002).

If patterns of nonlinear selection are preserved over long timescales (i.e., among related taxa), **γ** may also predict the structure of **D** if evolution occurs along “selective lines of least resistance” (Arnold et al., 2001; Schluter, 1996). Using the adaptive landscape metaphor popularized by Simpson (1953), this postulates that despite taxa evolving toward different (taxon‐specific) optima within a given lineage, peaks tend to be clustered in a ridge‐like arrangement on the landscape. This implies similarities among related species in the alignment of **γ**, possibly due to ecological demands that themselves show phylogenetic signal (Losos, 2008; Revell, Harmon, & Collar, 2008; Wiens & Graham, 2005).

Thus, one possibility is that both within‐ and among‐species covariance (**P** and **D**) will align with **γ**, underscoring the difficulty in disentangling the effects of selection on divergence from those of genetic constraints inferred from standing intraspecific trait variance (Conner, 2012). That is, the predictions of models emphasizing “genetic constraint” and “selection” are not mutually exclusive. Although separate lines of empirical study offer some support for intraspecific covariances (Brodie, 1992; Hunt, Blows, Zajitschek, Jennions, & Brooks, 2007; Revell et al., 2010; also see Roff & Fairbairn, 2012) or among‐species covariances (Hohenlohe & Arnold, 2008) corresponding with the pattern of nonlinear selection, we are not aware of an empirical study that has evaluated the concordance of among all three sets of parameters; that is, whether **γ** can simultaneously predict patterns of covariance observed at both micro‐ and macroevolutionary scales. In this study, we employed a genus of true bug (*Phymata*, Order: Reduviidae) to test the conjecture that patterns of both standing trait variation (in one species) and divergence among related taxa are explained by strong nonlinear sexual selection. We also discuss the implications of our findings with respect to the evolution of sexual dimorphism.

## 2 Methods

### 2.1 Background and study organisms

The genus *Phymata* comprises over 100 species and subspecies (Froeschner, 1988; Kormilev, 1962), with most occurring in the New World. For the purposes of this study, we do not distinguish between recognized species or subspecies and treat these as equivalent “taxa.” We should note that this treatment assumes no gene flow between taxa. Although we are unable to predict how introgression would influence among‐species covariance in trait means, a potential concern has been raised for an analogous issue with respect to the shape of the **G**‐matrices of interbreeding populations, whereby gene flow may inflate (co)variance along the direction of greatest difference between population means (Guillaume & Whitlock, 2007). The genus belongs to a basal and relatively old monophyletic clade in the Reduviidae (Hwang & Weirauch, 2012; Weirauch & Munro, 2009), but phylogenetic relationships at the species level have yet to be resolved.

**Figure 1:**
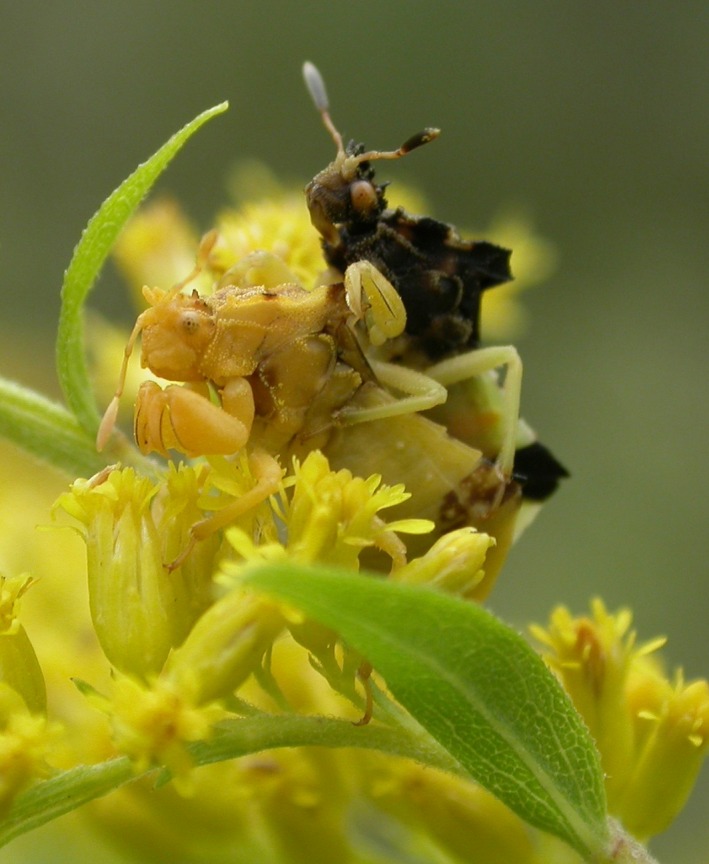
A male (upper) and female (lower) *Phymata americana americana* in precopulatory position

Ecologically, all *Phymata* are thought to be generalist sit‐and‐wait predators, and coloration may have been shaped by selection for crypsis (Schuh & Slater, 1995). Yet, sexual dimorphism in size and coloration appears to be widespread in the genus (Handlirsch, 1897; Kormilev, 1962; Melin, 1930) although quantitative treatments have only been performed for a handful of taxa (Mason, 1977; McLain & Boromisa, 1987; Punzalan, Rodd, & Rowe, 2008; Punzalan & Rowe, 2015). In one species (*P. americana americana*, Figure 1), we have previously detected multivariate sexual selection in a wild population, including negative nonlinear selection favoring intermediate values/combinations of melanic color patterns (Punzalan, Rodd, & Rowe, 2010). In this particular species, dark coloration appears to serve a thermoregulatory role in mediating male mating success (Punzalan, Rodd, & Rowe, 2008; Punzalan & Rowe, 2013; Punzalan, Rodd, & Rowe, 2008). A previous comparative study of sexual dimorphism among a much smaller subset of *Phymata* taxa suggested macroevolutionary conservatism of some components of male color pattern as well as biogeographical variation (i.e., across ‐species clines) consistent with melanism serving an important function in these taxa (Punzalan & Rowe, 2015).

### 2.2 Collection and rearing of *P. americana americana* for **P**‐matrix estimation

In 2013 and 2015, we collected 4th and 5th instar nymphs from a field site (Koffler Scientific Reserve, King, Ontario, Canada) housing a stable population of ambush bugs. Bugs were maintained individually in cages consisting of a transparent plastic tube (10.16 cm long, 1.27 cm diameter; 19.5 ml capacity) with a transparent rubber lid fitted with a flexible hole (Aquatube #53 floral watering tube). Nested within each tube was a semipermeable clear plastic spacer (2 cm long) that allowed air and small feeder insects to pass through. The tubes were mounted, lid down onto a feeding apparatus that provided bugs with live *Drosophila melanogaster* (Drosophilidae) and/or *Megaselia* sp. (Phoridae) per day (mean = 16.5, *SD* = 7.7; estimated from a separate assay). Every 4 days, cages were also provisioned with larger prey—either one adult or three 4‐ to 8‐d‐old pupae of Calliphoridae and/or Sarcophagidae, reared from ground beef. Every other day, bugs were checked for molts and the positions of cages on the feeding apparatus were randomized. All insects were maintained in a temperature‐controlled room at 25 ± 2°C, 25% RH, with a 14L: 10D fluorescent light cycle.

### 2.3 Museum specimens for **D**‐matrix estimation

Specimens were measured from the following museum collections: American Museum of Natural History, Canadian National Museum for Insects and Arachnids, Carnegie Museum of Natural History, National (Smithsonian) Museum of Natural History, Royal Ontario Museum, University of California Riverside Entomology Museum, and University of Michigan Museum of Zoology. Measured specimens were labeled with the name of the photographer (DP) and a unique four‐digit identification number.

### 2.4 Trait measurement, scaling, and standardization

In this study, we focus on three traits that are sexually homologous and often‐sexually dimorphic. Following established methodology, we measured pronotum width (PN)—a standard measure of overall size—as well as measures of mean dorsal darkness (MD) and mean lateral darkness (ML). The latter two traits are proxies of melanism of the integument in two distinct developmental units of the thorax (i.e., the pro‐ and mesothorax, respectively). Briefly, melanism was measured, using digital image analysis software, as the “value” (average number of black pixels) on a standardized patch of integument (see Punzalan, Cooray, Rodd, & Rowe, 2008, Punzalan & Rowe, 2015).

All three measured traits could be treated either as ratio or log‐interval scale types (Houle, Pelabon, Wagner, & Hansen, 2011). The types differ in their utility for biological interpretation but are also accompanied by limitations in representing variability in a given dataset. For example, both provide valid representations regarding the relative variability of traits; however, the latter assumes that differences in trait values are not of interest (Hansen & Houle, 2008; Houle et al., 2011). Given the difficulty in making a priori assumptions regarding the importance of such differences, we performed tests of concordance among matrices and/or their eigenvectors using both mean‐standardized data and the raw, natural log‐transformed data. Mean standardization has several properties that are desirable for this study, including appropriate scaling of variability of each trait by its absolute size (Houle et al., 2011) while also accommodating differences in trait means, corresponding to slight differences in methodology used to obtain data for each of the three matrices. For example, although methods were always internally consistent within each dataset, trait measures for **γ** and **P** were obtained using methods optimized for measurement of live bugs (albeit in different years) while **D** was measured from preserved specimens mounted on entomological pins. For estimation of the former two matrices, phenotype data was standardized by trait means within each sample (i.e., the mean of *P. americana americana* estimated from the field mating success study and each of the two common‐garden studies, respectively); for the latter, phenotypic data were divided by the trait means derived from *P*. *americana americana* obtained using the protocol for museum specimens. That is, comparisons among selection coefficients, intraspecific variability, and interspecific divergence are conducted in units of *P. americana americana* trait means.

Log‐transformation of data is often adopted on account of a number of well‐known statistical benefits. In the present dataset, however, log‐transformation actually had undesirable effects (i.e., generating skew in already normally distributed phenotypic data), which may have contributed to some problems encountered in some analyses of this dataset (e.g., **D**‐matrix estimation using a mixed model approach; discussed below).

In all analyses, results using both mean‐standardized and natural log‐transformed data were qualitatively identical. Consequently, we primarily focus on the analyses using mean‐standardized estimates except for a few key statistical tests, for which we present the results from both sets of analyses.

### 2.5 Estimating phenotypic selection

Phenotypic selection gradients were derived from a previous field study of sexual in a wild population of *P*. *americana americana* (Punzalan et al., 2010). Using the original digital photographs from the prior study, we obtained measures of MD and ML and, following the conventional method, estimating selection gradients based on the partial regression coefficients of relative fitness on the variance‐standardized phenotype distribution (Brodie, Moore, & Janzen, 1995; Lande & Arnold, 1983; Stinchcombe, Agrawal, Hohenlohe, Arnold, & Blows, 2008). We report the conventional variance‐standardized selection gradients for the homologous traits (PN, MD and ML) in Table 1 but convert these to mean‐standardized gradients (Hereford, Hansen, & Houle, 2004) in corresponding comparisons with **P** and **D** (discussed in detail below).

**Table 1:** Trait distributions for male *Phymata* from museum collections

Taxon	*N*	PN	MD	ML
*P. acuta*	5	1.93 (0.07)	205.35 (14.12)	221.30 (7.59)
*P. albopicta*	6	2.19 (0.05)	184.59 (8.43)	188.08 (10.44)
*P. americana americana*	140	3.10 (0.21)	236.13 (19.46)	213.40 (38.12)
*P. americana coloradensis*	62	3.37 (0.23)	211.89 (29.54)	147.22 (34.39)
*P. americana metcalfi*	82	2.80 (0.19)	230.57 (26.82)	110.93 (19.25)
*P. arctostaphylae*	8	3.10 (0.13)	253.40 (9.44)	216.27 (11.71)
*P. armata*	6	2.28 (0.14)	182.26 (13.79)	177.44 (12.42)
*P. bimini*	6	2.67 (0.09)	219.96 (21.90)	118.72 (21.02)
*P. borica*	10	2.64 (0.22)	240.11 (16.38)	148.43 (26.47)
*P. carinata*	6	3.37 (0.23)	237.03 (24.01)	128.41 (5.30)
*P. chilensis*	10	3.14 (0.15)	226.21 (28.46)	115.99 (18.54)
*P. communis*	5	2.59 (0.13)	237.67 (14.91)	132.27 (6.26)
*P. crassipes*	6	2.74 (0.10)	230.98 (9.51)	177.97 (17.28)
*P. delpontei*	5	2.38 (0.04)	188.34 (25.78)	170.63 (19.00)
*P. fasciata fasciata*	38	3.07 (0.22)	186.61 (27.05)	163.33 (26.58)
*P. fasciata mexicana*	7	3.11 (0.16)	217.15 (15.06)	181.59 (24.12)
*P. fasciata panamensis*	4	3.29 (0190)	222.41 (27.05)	171.36 (16.16)
*P. fortficata argentina*	9	3.70 (0.19)	205.77 (11.91)	187.74 (14.92)
*P. fortificata fortificata*	9	4.04 (0.28)	192.77 (25.98)	189.18 (24.83)
*P. granulosa*	6	3.35 (0.32)	253.23 (9.66)	209.89 (26.17)
*P. guerini*	7	2.97 (0.24)	201.19 (16.23)	145.83 (16.05)
*P. lindigiana*	6	1.87 (0.08)	235.57 (10.57)	136.71 (8.66)
*P. luxa*	6	1.91 (0.15)	179.80 (13.70)	173.70 (15.28)
*P. marginata*	8	2.17 (0.09)	196.83 (8.76)	156.73 (21.82)
*P. monstrosa*	5	2.67 (0.17)	246.32 (14.64)	206.51 (34.22)
*P. mystica*	55	3.15 (0.25)	209.69 (37.69)	176.76 (45.31)
*P. nouahlieri*	5	2.28 (0.06)	186.64 (21.75)	194.61 (19.24)
*P. pacifica pacifica*	10	2.61 (0.15)	164.92 (35.22)	131.51 (16.05)
*P. pacifica stanfordi*	6	2.58 (0.18)	210.43 (34.32)	116.81 (19.67)
*P. pennsylvanica*	143	2.76 (0.18)	226.31 (23.05)	213.81 (29.22)
*P. praestans*	8	4.19 (0.11)	215.09 (17.96)	138.95 (11.70)
*P. rossi*	7	2.84 (0.16)	212.57 (12.52)	135.62 (9.26)
*P. salicis*	7	2.50 (0.11)	160.51 (27.88)	119.79 (12.48)
*P. severini*	6	2.21 (0.09)	218.31 (15.95)	203.70 (28.05)
*P. simulans*	5	2.03 (0.12)	235.62 (13.18)	184.18 (40.65)
*P. stali*	13	2.55 (0.10)	204.85 (30.43)	154.59 (30.00)
*P. vicina*	52	2.19 (0.19)	221.68 (25.27)	182.00 (31.92)

Mean and standard deviations (in parentheses) for pronotum width (PN) in mm, average darkness for mean dorsal (MD), and mean lateral melanism (ML) measured in units of (average) number of pixels. *N* is sample size

The matrix of nonlinear selection gradients (**γ**) was diagonalized to find the canonical axes (*m*) of nonlinear selection (Blows & Brooks, 2003; Phillips & Arnold, 1989), and significance testing of curvature was performed using a randomization procedure to derive a null distribution of eigenvalues for eigenvectors calculated from the permuted data (Reynolds, Childers, & Pajewski, 2010). We interpreted the eigenvector with the largest negative eigenvalue to represent the direction of strongest multivariate stabilizing selection (*m*_max_). Although we perform subsequent analyses using the explicitly multivariate approach advocated by some (Blows, 2007; Walsh & Blows, 2009), the eigenvectors of nonlinear selection were closely aligned with original trait space (i.e., approximately stabilizing selection on MD), permitting a relatively straightforward biological interpretation (Conner, 2007).

### 2.6 Estimating intraspecific trait covariation, the **P**‐matrix

The **P**‐matrix was estimated from wild‐caught subadults (nymphs), maintained under common‐garden conditions in the laboratory through adulthood, in two separate years (see Section 2, Collection and rearing of *P. americana americana* for **P**‐matrix estimation) for information on rearing conditions). Although body size (PN) is fixed upon adult emergence, we maintained bugs until 14d of (adult) age because dark coloration traits may take up to 2 weeks to reach their asymptotic values (Punzalan, Cooray et al., 2008). Subsequently, we sexed and photographed bugs for later measurement of the three traits. In total, 50 males in year 1 and 58 males in year 2 were available for the estimation of the **P**‐matrix. Standard errors of each element were estimated using a delete‐one jackknife approach (Manly, 1997) in R (http://www.R-project.org) using the package “bootstrap” version 2015.2 (Efron & Tibshirani, 1993). For mean‐standardized comparisons involving **P**, we conducted analyses using data pooled between years (but standardized separately within year to remove differences in multivariate means), as well as when considering **P**‐matrices separately. Results were remarkably consistent irrespective of whether we used pooled data or analyzed **P** separately by year (i.e., **P**‐matrices in the 2 years were very similar; data not shown). For simplicity, we report the results from the pooled mean‐standardized data.

### 2.7 Estimating among‐species covariation, the **D**‐matrix

We employed a straightforward approach (see Hohenlohe & Arnold, 2008; Schluter, 1996) to estimating the **D**‐matrix. We calculated **D** as the variance/covariance matrix among trait means for males of 37 taxa, obtained from photographs of preserved museum specimens (*N *=* *779, Table 1), including those from a previous study (Punzalan & Rowe, 2015). Many *Phymata* spp. are rarely collected or identified, so sample sizes for each taxon varied depending on availability of determined material in museum collections. For the present purposes, we included taxa for which we had a complete set of trait measurements for a minimum of four males (median = 7). We should point out that, for some datasets, **D** can be estimated using a multivariate mixed model (e.g., McGuigan, Chenoweth, & Blows, 2005; Schoustra, Punzalan, Dali, Rundle, & Kassen, 2012), by treating species as a random effect and estimating the species‐level covariance matrix. A benefit of the mixed model approach is that it allows for estimates of uncertainty in the elements of **D**. We employed this approach as well, using restricted maximum likelihood in the MIXED procedure in SAS (v. 9.2, Cary, NC) to estimate **D** for the mean‐standardized traits. It was not possible to derive such estimates using the natural log‐transformed dataset because models failed to converge.

### 2.8 Measuring concordance among the three matrices and significance testing

First, we tested for similarity between **P** and **D**, using the Flury (1988) hierarchy and implemented using the common principal components analysis program (Phillips & Arnold, 1999). CPCA has the ability to distinguish matrix concordance of various degrees, ranging from matrix equality, proportionality, concordance of (some) eigenvectors to unrelated matrices. To find the most probable model of matrix similarity, we employed both the model building (based on the lowest score of Akaike's Information Criterion) and “jump‐up” (based on pair‐wise model comparisons) (Phillips & Arnold, 1999).

To test the degree to which nonlinear (in this case, negative) selection shapes intraspecific variation, we compared the alignment *m*_max_ (the direction of greatest negative nonlinear selection in **γ**) with the eigenvector associated with the least variability in **P**, or *p*_min_ (the direction of least trait variance/covariance), by means of the absolute value of the inner product (or vector correlation, ρ) between *m*_max_ and *p*_min_. As the (inverse of the) phenotypic covariance matrix is used in the estimation of **γ**, one concern is that this could generate spurious correlations with **P**. However, in this study, **P** was estimated separately from the selection study, so an observed correlation cannot be strictly an artifact of this mathematical relationship.

For each sample of **P** (i.e., year), significance testing was performed using a permutation test, whereby observed trait values from the **P**‐matrix dataset were shuffled (within traits) prior to calculation of a random **P**‐matrix and its associated eigenvectors. The permutation test assessed the likelihood of obtaining the observed (or greater) absolute value of ρ by chance (10,000 iterations), given the multivariate phenotype distribution. The same procedures were used to perform the analogous test, comparing *m*_max_ with the direction of least across‐species multivariate divergence (*d*_min_). These analyses are similar to the approach taken by other authors (e.g., Hohenlohe & Arnold, 2008; Schluter, 1996) in that the reference vector (*m*_max_) is not allowed to vary and, thus, implicitly assumes this direction to be without error. We also caution that a potential bias arises in comparisons of **P** and **γ**, stemming from the errors in estimation of the latter, that causes the dominant (canonical) vectors of nonlinear selection to tend toward alignment with dimensions of low phenotypic variance (Morrissey, 2014). An alternative approach is to use projection pursuit regression to estimate the vector (projection) that explains the most covariance with relative fitness (Morrissey, 2014; Schluter & Nychka, 1994). In this study, no significant linear selection was observed, allowing for a relatively straightforward interpretation of the projection as a direction that describes (stabilizing) nonlinear selection (see Results). We calculated this projection using the program provided by D. Schluter (https://www.zoology.ubc.ca/~schluter/wordpress/software/), over a range of possible smoothing parameters (λ = −10 to λ = 10 in increments of 2) with 5,000 random projections. Coordinates of the projection were invariant throughout almost the entire tested range (i.e., for all λ > −10). To our knowledge, the potential alignment bias does not apply to the comparisons of **γ** and **D**.

Note that, our approach to comparing alignment of eigenvectors differs from the approach used by some previous studies (e.g., Blows, Chenoweth, & Hine, 2004; Hohenlohe & Arnold, 2008; Revell et al., 2010) that compared the direction of weakest selection (i.e., **ω**_max_ **,** the leading eigenvector of the negative inverse of **γ**) with the direction of most variability (e.g., *g*_max_ *, p*_max,_ or *d*_max_). Our rationale for essentially doing the reverse (i.e., aligning the axis of strongest selection with the axes of least variation) is that it provides a more direct test of the prediction that variance will be most constricted in the principal direction of stabilizing selection. The utility of the minor eigenvectors has been recognized by other authors in analogous evaluations of **D**‐matrices (Stock, Campitelli, & Stinchcombe, 2014). Our alignment approach is also advantageous in that we employ the direction on the estimated selection surface that has the most statistical support (e.g., eigenvectors associated with low eigenvalues could simply reflect low statistical power in a selection analysis rather than convey biological information). We emphasize that our approach does not assume that our estimates of phenotypic sexual selection are necessarily reflections of *net* selection. That is, we find it unrealistic to expect stabilizing sexual selection to predict where all/most variation and divergence ought to lie—surely, a considerable amount of evolution is expected to reflect selection via other (unmeasured) fitness components. Instead, our focus is on identifying whether stabilizing sexual selection has contributed to restricted relative variability as predicted by theory.

We also employed a second approach, directly estimating the length (*e*) of the projection **P** and **D**, respectively, onto the space defined by each eigenvector of **γ**, and computed as

$$e\left(m_{i}\right)=m_{i}^{\prime }\;A\;m_{i},$$

where *m* is the eigenvector of the *i*th direction of **γ**, ‘indicates transpose, and **A** is a matrix representing **P** or **D**, accordingly. Note that *e(m*_*i*_ *)* is equivalent to the variance along a particular direction and is analogous to the measure of evolvability proposed by Hansen and Houle (2008, eq. 1). However, given that in this study, the vectors of interest do not describe directional selection and predicted evolutionary response, *e(m*_*i*_ *)* should be strictly interpreted as a measure of variance in **P** and **D** defined in the *i*th direction of nonlinear selection. For both intra‐ and interspecific data, we compared *e* in the direction of strongest nonlinear selection, *e*(*m*_max_) to estimates in the other two orthogonal directions of nonlinear selection, *e*(*m*_1_) and *e*(*m*_2_). We also compared these to an average *e* from 1,000 random directions, using the “MeanMatrixStatistics” function in the EvolQG package in R (Melo, Garcia, Hubbe, Assis, & Marroig, 2015). This differs from the alignment approach in that it quantifies the amount of variance (i.e., vector norm or magnitude) in a given direction—the alignment approach is unconcerned with magnitude of variance but on the angle between unit vectors (which, in this case, describe dimensions of relative variability and the relative strength of selection).

Additional statistical analyses and mathematical operations were carried out using JMP^®^ version 4.0.3 (SAS Institute) and the R base package (http://www.R-project.org).

We acknowledge that our analyses of concordance between **D** and the other two matrices are limited by a lack of phylogenetic information and our analyses essentially assume a “star” phylogeny. This can potentially result in a conflation between the effects of the actual divergence rate matrix and coancestry (see Revell & Harmon, 2008) on the observed **D**‐matrix. We address these limitations in more detail in Section 4.

## 3 Results

### 3.1 An adaptive ridge generated by sexual selection

**Figure 2:**
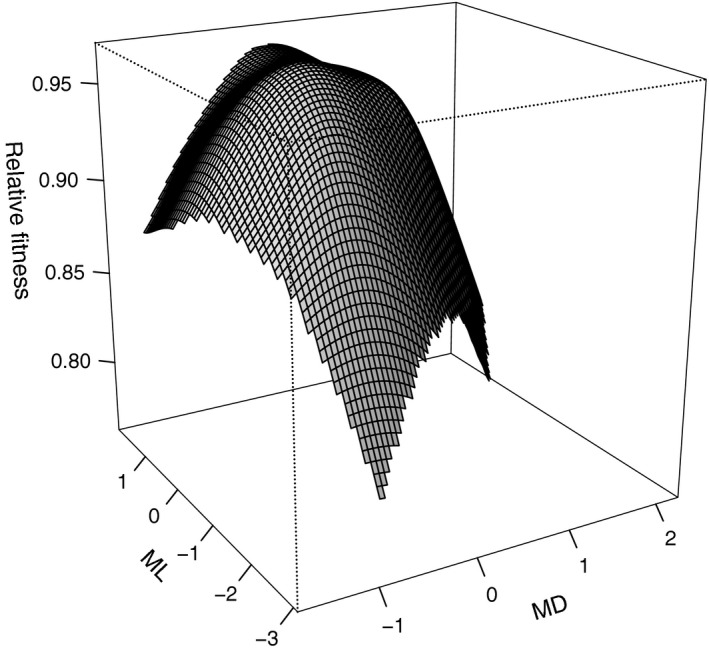
Thin plate spline depiction of the phenotypic (sexual) selection surface estimated from male mating success for a sample from a wild population of *Phymata americana*. Traits are mean dorsal darkness (MD) and mean lateral darkness (ML) measured in units of (average) number of pixels, each variance‐standardized according to the phenotypic distribution of males in the sample

Phenotypic selection analyses of sexual selection on three traits in *Phymata americana americana* indicated no significant linear selection, but moderate‐to‐strong nonlinear selection. In original trait space, this pattern was manifested primarily as negative nonlinear selection on MD, with some of stabilizing selection on ML, as well as positive correlational selection on MD and ML (Table 2, Figure 2). For MD, the local maximum was within the range of the phenotypic distribution, indicating that the quadratic selection gradient was indeed approximating true stabilizing selection (sensu Mitchell‐Olds and Shaw 1987). Canonical analyses identified negative nonlinear selection acting primarily on the eigenvector *m*_3_, hereafter referred to as *m*_max_ (λ = −0.522, permutation test: *p = *.0319). Irrespective of data transformations, the loadings of *m*_max_ describe an axis that primarily separates MD from PN and ML (Table 3). Thus, the canonical selection surface was quite closely aligned with the original trait space with most curvature stemming from selection on MD, or possibly on a linear combination of MD and ML (Figure 2).

**Table 2:** Variance‐standardized linear (β) and nonlinear (γ) sexual selection gradients estimated for three traits in *P. americana americana*. Trait abbreviations and units of measurement are the same as in Table 1

	β	γ		
		PN	MD	ML
PN	0.008 ± 0.084	0.137 ± 0.133		
MD	−0.029 ± 0.090	0.005 ± 0.145	−0.365 ± 0.185^a^	
ML	0.021 ± 0.021	−0.118 ± 0.158	0.214 ± 0.156	−0.206 ± 0.199

Linear model: multiple *R* ^2^ = .003, *F*_3,40_ = 0.038, *p* = .989.

Nonlinear model: multiple *R* ^2^ = .67, *F*_9,34_ = 0.911, *p* = .527.

a *p* = .053.

**Table 3:** Linear (θ) and nonlinear selection (λ) gradients along each canonical vector (*m*) of the variance‐standardized γ‐matrix (see Table 1 for trait abbreviations)

	θ	λ	PN	MD	ML
*m*_*1*_	0.004	0.181	0.926	−0.131	−0.354
*m*_*2*_	0.001	−0.094	0.361	0.581	0.729
*m*_*3*_	0.037	−0.522^a^	0.111	−0.803	0.585
					

a Statistical significance at α = 0.05 estimated from multiple regression (linear) or permutation tests (nonlinear). Abbreviations and units are the same as in Table 1.

### 3.2 Alignment of phenotypic variance and divergence with the adaptive ridge

**Figure 3:**
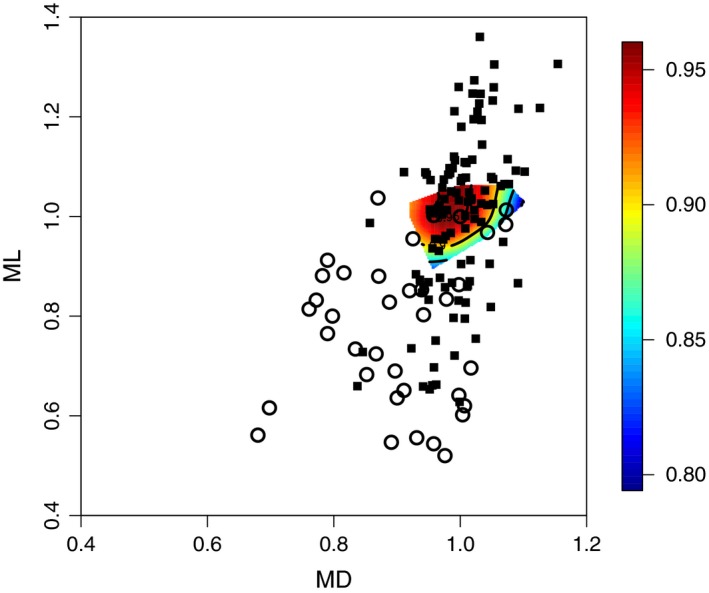
The individual male phenotypic values (i.e., intraspecific variation; filled squares, *N *=* *108) from the common‐garden study and the taxon mean values (i.e., interspecific variation; open circles, *N *=* *37) superimposed on the contour plot depicting the fitness surface of *P. americana americana* in (mean standardized) two‐trait space. Trait abbreviations are the same as in Figure 2. Heat map contours indicate estimated relative fitness

Common principal components analysis using mean‐standardized data revealed that **P** and **D** (Table 4) did exhibit significant similarity; the model‐building procedure indicated that “common eigenvectors” are the best model, although the “jump‐up” approach supported matrix proportionality (Table 5). When using the natural log‐transformed data to estimate **P** and **D** CPCA analyses indicated identical conclusions (not shown). Comparisons among all three matrices indicated that the eigenvector describing the most convex (negative nonlinear) selection, *m*_max_, was significantly aligned with the eigenvector capturing the direction of least standing phenotypic variance in *P. americana americana*,* p*_min_ (mean standardized: ρ = 0.78, log‐transformed: ρ = 0.74, both *p *<* *.0001), consistent with stabilizing sexual selection that has diminished standing variance in a predictable manner (i.e., mostly on dorsal melanism, MD; Figure 3). Similarly, the direction of least among‐species divergence, *d*_min_, was significantly aligned with *m*_max_ (mean standardized: ρ = 0.82, *p *=* *.0010; log‐transformed: ρ = 0.79, *p = *.1325), indicating that multivariate divergence was most restricted in the direction of strongest stabilizing sexual selection. Performing matrix comparisons with **P** estimated separately by year did not alter these conclusions (data not shown). We also verified that the multivariate direction described by *m*_max_ (i.e., using the canonical approach) had very similar vector coordinates to the one obtained by the projection pursuit approach (vector correlation of mean‐standardized data = 0.92). Compared to other directions, *e*(*m*_max_) did not show marked differences from other directions, including random directions (Table 6). This suggests that although sexual selection seems to have restricted variability along *m*_max_, there still exists substantial phenotypic variance and interspecific divergence in this direction, comparable to other phenotypic directions.

**Table 4:** The **P**‐matrix estimated from 108 *Phymata americana americana* males reared under common‐garden conditions (A) and the D‐matrix based on the trait means for 37 *Phymata* taxa when estimated directly as the covariance among species means (B) versus from a mixed model (C). Trait data is standardized by mean values of *P. americana americana* and, to facilitate readability, **P** is multiplied by 100 and **D** is multiplied by 10. Diagonal elements represent the variances and off‐diagonals represent the covariances. In (A) and (C), values in parentheses indicate standard errors corresponding to each matrix element. Abbreviations and units are the same as in Table 1

	PN	MD	ML
(A) **P**‐matrix			
PN	0.243 (0.034)		
MD	0.016 (0.026)	0.265 (0.048)	
ML	−0.063 (0.075)	0.421 (0.096)	2.717 (0.340)
(B) **D**‐matrix (species means)			
PN	0.335		
MD	0.036	0.100	
ML	−0.017	0.026	0.236
(C) **D**‐matrix (mixed model)			
PN	0.308 (0.083)		
MD	−0.007 (0.033)	0.095 (0.028)	
ML	−0.033 (0.057)	0.045 (0.034)	0.334 (0.078)

**Table 5:** Results of common principal components analysis of matrix similarity between the mean‐standardized **P**‐ and **D**‐matrices, using two approaches

Comparison in Flury hierarchy	Model building			Jump‐up		
	χ^2^	*df*	AIC	χ^2^	*df*	*p*‐Value
Equality	104.95	1	150.86	150.86	6	<.0001
Proportionality	45.17	2	47.91	45.91	5	<.0001
CPC	0.65	1	6.74	0.74	3	.8638
CPC1	0.09	2	8.09	0.09	2	.9551
Unrelated	–	–	–	–	–	–

The “model building” approach tests the model in each line against the model indicated on the line below it. The “jump‐up” approach tests each model against a model of “Unrelated.” Abbreviations *df* and *AIC* refer to degrees of freedom and Akaike's Information Criterion, respectively.

**Table 6:** Measures of variance (*e)* along the eigenvectors of the mean‐standardized **γ** are as follows: *m*_max_ *= *[0.128, −0.686, 0.716]*, m*_2_ *= *[0.922, −0.184, −0.341], and *m*_1_ = [0.365, 0.704, 0.609], estimated from the mean‐standardized **P**‐ and **D**‐matrices (i.e., Table 4A and B)

	**P**‐matrix	**D**‐matrix
*e*(*m*_max_)	0.011	0.014
*e*(*m*_2_)	0.006	0.032
*e*(*m*_1_)	0.015	0.021
Average *e*	0.010	0.023

Average *e* is the mean value from 1,000 random directions of *m*. Coordinates of m‐vectors correspond to loadings on PN, MD, and ML (abbreviations the same as in Table 1).

## 4 Discussion

We compared a set of micro‐ and macroevolutionary parameters for *Phymata* to address nonexclusive explanations for two broadly observed and puzzling phenomena: the prominence of relatively restrained divergence (including phenotypic stasis) within a lineage and proportionality between intra‐ and interspecific trait covariation. Our results illustrate a simple resolution, based on the predicted effects of stabilizing selection at both short and long evolutionary timescales. We discuss our findings with respect to the potential role of sexual selection in shaping divergence in this group of insects, and in light of the ongoing debate regarding the relative importance of selective and genetic constraints.

### 4.1 Stabilizing selection aligned with variation within‐ and between‐species

We confirmed the first prediction, that standing phenotypic trait covariance in *P. americana americana* conforms to the pattern of nonlinear selection, exhibiting a characteristic reduction of trait variance in the direction of strongest stabilizing selection. This microevolutionary expectation derives from the effects of within‐generation selection on the genetic variance–covariance matrix (Phillips & Arnold, 1989) and/or a developmental system that interacts with the environment in a manner that preserves beneficial trait combinations (Cheverud, 1996; Marroig & Cheverud, 2001). The similarity between **P** (or **G**) with **γ** has been corroborated in several other studies (Brodie, 1992; Conner & Via, 1993; Revell et al., 2010), although fewer have performed so with respect to sexual selection (Hunt et al., 2007; McGlothlin, Parker, Nolan, & Ketterson, 2005).

We also found support for the second prediction that macroevolutionary divergence, represented by the **D**‐matrix, was most restricted along the principal axis of stabilizing sexual selection, approximating a selective line of least resistance. We believe this result might account for phenotypic conservatism in some secondary sex characters (i.e., male melanism) in *Phymata*. Our knowledge regarding the pattern of sexual selection is restricted to (and estimated for) a single species (i.e., *P*. *americana americana*), which,undoubtedly, is an imperfect characterization of the landscape for all taxa. Over the history of a lineage, any number of unidentified microevolutionary processes are expected to result in peak displacements. Nonetheless, the data suggest that these displacements occur within a relatively restricted range, fully consistent with many models of macroevolution (e.g., an Ornstein‐Uhlenbeck process; Hansen, 1997) and consistent with Simpson's (1953) notion of fitness peaks remaining within an adaptive zone. There is some empirical evidence consistent with adaptive landscapes remaining stable for long enough periods to generate repeatable and predictable patterns of phenotypic divergence (e.g., Mahler, Ingram, Revell, & Losos, 2013). A more direct evaluation of this conjecture (requiring the estimation of **γ** in multiple, related taxa; Arnold et al., 2001) is a challenging feat and beyond the scope of the present study.

We acknowledge a number of limitations and concerns presented by our dataset; first, our estimates of standing variance employed **P** instead of **G** to illustrate the potential alignment of phenotypic variance with relevant descriptors of selection and divergence, and this is a well recognized issue of contention. The use of **P** in the present study is purely a consequence of logistic difficulties (i.e., multigenerational, mass rearing) associated with our study system that currently precludes the estimates of **G**. While we agree that we must be cautious about our interpretation of **P**, our data are simply descriptors of an existing pattern of intraspecific covariation and we show that this appears to be aligned with other relevant evolutionary parameters. It is certainly possible that, in reality, **P** does not resemble the underlying **G**‐matrix, but if so, it is not clear how environmental (or nonadditive genetic) sources of covariance should generate the alignment we observe.

Second, we rely on estimates of **P** obtained for a single species. As with estimates of **γ**, ideally one would have estimates of **P** for all species and analyses of alignment with **D** could be performed using the among‐species average **P** (or **G**) matrix as a more accurate estimate of the (time) average **G**‐matrix, upon which the neutral expectation for divergence under drift holds even with fluctuating variances (see Hohenlohe & Arnold, 2008; Lande, 1979). Additionally, multiple estimates of **P** could be used to evaluate several related questions including their degree of with species‐specific selection gradients (Arnold et al., 2001), as well as the rate and direction of divergence among species in intraspecific covariation (e.g., Berner, Stutz, & Bolnick, 2010; Steppan et al., 2002).

A third point of concern is that **γ** has a tendency to align with **P**, generating potentially spurious statistical concordance between eigenvectors of the respective matrices (i.e., *p*_min_ and *m*_max_), when using the orthogonal approach embraced by most previous authors (Blows & Brooks, 2003; Phillips & Arnold, 1989; see Morrissey, 2014). The degree to which this will always pose a problem in interpreting empirical data is not clear, but one approach is to employ alternative methods to estimating the major axes of **γ**. In the present study, we compared the principal directions obtained by canonical and projection pursuit approaches and found strong agreement between the two, suggesting that the alignment we describe was not simply an artifact of the canonical approach.

Fourth, the absence of a phylogeny for these taxa prevents analyses that account for coancestry, which itself can impose covariance in **D**. Phylogenetic data (using **D** from independent contrasts; for example, Baker & Wilkinson, 2003; Revell, 2007; Revell et al., 2007) or by incorporating phylogeny directly into the hypothesis tests of divergence (Hohenlohe & Arnold, 2008; Revell & Harmon, 2008) can be used to evaluate whether the pattern of divergence is seen in *Phymata* differs from expectations under a neutral model of mutation–drift (Lande, 1979; Lynch & Hill, 1986). An emerging empirical consensus, however, is that genetic drift is a poor explanation for patterns of divergence over most timescales (Arnold, 2014). Although we could not directly evaluate various selection‐based models for divergence (e.g., reviewed in Estes & Arnold, 2007), we stress that our results are not consistent with a model with simply random movement of adaptive peaks (e.g., Brownian motion of the optimum). Instead, the characteristic covariance among phenotypic means suggests nonindependence in the movement of peaks, although it is unclear what forces generate this covariance and to what degree this reflects the temporal and hierarchical component of divergence (i.e., phylogeny).

Numerous sources of stabilizing selection, including ecological, developmental, and physical limits (Arnold, 1992; Estes & Arnold, 2007), have been proposed to set the boundaries to adaptive zones, and here, we highlight how these might include sexual selection. Familiar examples of stabilizing sexual selection constraining divergence can be found in systems characterized by mate choice acting on sexual signals. For example, Brooks et al. (2005) estimated the surface representing female preference for male call characters in the field cricket *Teleogryllus commodus*, finding that mean phenotypes for four populations were indeed very close to the adaptive peak. Naturally, mate choice is not the only mechanism that can generate stabilizing sexual selection; mating in *Phymata* spp. appears to resemble scramble polygyny in which mate guarding is very effective and male mate searching performance is a main determinant of male mating success (Dodson & Marshall, 1984; McLain & Boromisa, 1987; Punzalan, Rodd, & Rowe, 2008; Punzalan, Rodd, & Rowe, 2008). The pattern of sexual selection also appears to depend on demographic parameters (e.g., sex ratio and density; Punzalan et al., 2010) as well as microclimatic variables (Punzalan & Rowe, 2013), presumably because of how it mediates the efficacy of searching. Thus, our results are suggestive of some degree of conservatism in aspects of both the mating system and ecology of *Phymata*.

### 4.2 Sexual selection as an agent of stasis

Darwin (1871) postulated sexual selection largely to explain the exaggeration and diversification of secondary sex characters seen within lineages. Subsequently, authors have come to view sexual selection primarily as an agent of diversification (Andersson, 1994). While the importance of sexual selection for generating phenotypic diversity is not in doubt, our analyses offer a counterpoint, underscoring that (as Darwin recognized) sexual selection does not fundamentally differ from other forms of selection. Specifically, we show that it can contribute to an observed instance of evolutionary stasis, at least in the broad sense of “restrained” divergence within a clade (sensu Futuyma, 2010). In *Phymata*, males exhibit relatively lower degrees of divergence in MD than do their female counterparts (also see Punzalan & Rowe, 2015), consistent with prolonged periods of stabilizing sexual selection on this male trait. Fittingly, one of the earliest studies to report male‐biased conservatism was Wallace's (1865) treatment of sexual dimorphism in mimetic *Papillio* butterflies, for which later authors have invoked sexual selection and species recognition as possible selective constraints on male coloration (Kunte, 2008).

**Figure 4:**
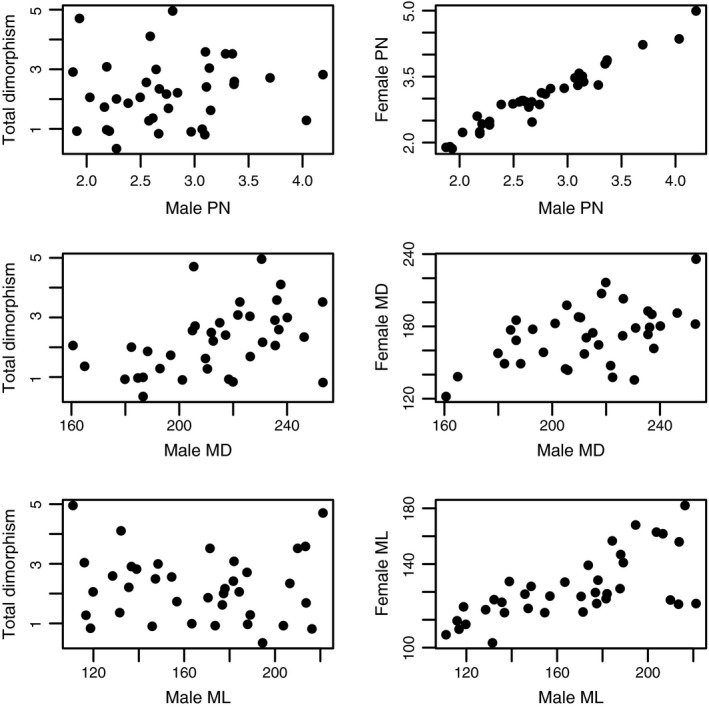
Bivariate plots depicting contributions of divergence in male traits to an index of total (multivariate) sexual dimorphism (*d*_*i*_) (left panels) and the among‐species covariance between male and female homologous traits (right panels). Each row corresponds to plots for each trait separately. The index *d*_*i*_ was computed as the male–female Mahalanobis distance for mean‐standardized phenotypes considering the three measured traits. Methods for trait measurement of females were identical to those used for males (see Section 2). Sample sizes for females ranged from 2 to 106 (median = 7). PN is measured in mm, and MD and ML are in units of average pixel number (i.e., darkness). Total dimorphism is unit free

We emphasize that stasis and diversification are not mutually exclusive when taking an appropriate, multivariate view of phenotypic evolution. For example, within a lineage, one trait might be best characterized by evolutionary stasis while a second trait may exhibit very high levels of diversity. Indeed, elements of the **D**‐matrix for *Phymata* (Table 4) indicate dorsal melanism exhibits relatively low coefficients of variation among taxa but considerable among‐species diversity in lateral melanism, and even higher diversity for the index of body size. Nevertheless, even for the trait exhibiting the lowest relative divergence (MD), taxa still vary considerably in their degree of sexual dimorphism. In fact, among‐species variance in an index of multivariate sexual dimorphism (i.e., *d*_*i*_ in Punzalan & Rowe, 2015; based on Mahalanobis distance) largely reflects divergence in male dorsal color darkness, and concomitant evolution in (the opposite direction on) females (Figure 4). This is consistent with prior studies pointing to melanism as a principal target of divergent sex‐specific selection (e.g., Punzalan & Rowe, 2015; Punzalan, Rodd, & Rowe, 2008). Furthermore, these data suggest that sexual dimorphism evolves readily despite positive male–female macroevolutionary correlations (Figure 4) potentially arising from sexually concordant expression of genetic variance (Baker & Wilkinson, 2001; Lande, 1980; Reeve & Fairbairn, 2001). Clearly, selective constraints on a given trait (or trait combination) do not necessitate constraints on sexual dimorphism itself.

### 4.3 Alignment of genetic and selective lines of least resistance

One of the most debated subjects in evolutionary biology is with regard to the importance and persistence of genetic constraints. While genetic constraints must exist at a fundamental level (Gould & Lewontin, 1979), many have questioned whether estimates of standing trait variability have any importance for evolutionary response over the long term (Conner, 2012; Futuyma, 2010; Pigliucci, 2006). Empirical data are somewhat equivocal, with some studies of among‐population/species divergence apparently biased by trait covariance (e.g., Baker & Wilkinson, 2003; Bégin & Roff, 2003, 2004; Hunt, 2007; McGuigan et al., 2005; Schluter, 1996), but also many empirical studies finding limited predictive power for standing genetic (Berner et al., 2010; Hohenlohe & Arnold, 2008; Merilä & Björklund, 1999) and mutational (Schoustra et al., 2012) variation for determining patterns of divergence.

In the present study, we found that intra‐ (**P**) and interspecific variation (**D**) were, in fact, concordant but also that both were closely aligned with the features of the adaptive landscape. That is, our findings are consistent with intra‐ and interspecific variation being shaped by similar selective processes (i.e., nonlinear selection). An analogous conclusion comes from a theoretical study that predicts “triple alignment” between mutational and genetic variances with the pattern of stabilizing selection (Jones, Bürger, & Arnold, 2014; also see Jones, Arnold, & Bürger, 2007, Lande, 1980). It is tempting to ascribe such results to the primacy of selection over genetic (including mutational) constraints at both micro‐ and macroscales. However, “selection” and “genetic constraints” are, of course, not exclusive alternatives. We show here that, even though stabilizing selection appears to explain the pattern of restricted trait covariation within‐ and between‐species, this does not preclude the existence of substantial evolutionary potential within this boundary [i.e., *e*(*m*_max_)]. Bolstad et al. (2014) reported evidence of divergence principally restricted to directions that mirror the pattern of standing trait covariance, despite apparently ample evolvabilities in any given direction. Undoubtedly, trait (i.e., genetic and especially mutational) covariance imposes constraints on evolutionary response and the timescales over which this covariance is preserved is an open issue—but if the metric used to estimate genetic constraint (i.e., standing variation) itself conforms to the adaptive landscape, then the distinction between the two becomes blurred. At the very least, we believe our results highlight the need for caution when attempting to interpret standing patterns of trait variation necessarily as indicators of past and future macroevolutionary constraints.

## Conflict of Interest

The authors declare no conflict of interest.
